# Primary Myeloid Sarcoma Masquerading as an Obstructing Duodenal Carcinoma

**DOI:** 10.1155/2012/490438

**Published:** 2012-11-29

**Authors:** Preeti Narayan, Vijayashree Murthy, Mu Su, Rosemonde Woel, I. Robert Grossman, Ronald S. Chamberlain

**Affiliations:** ^1^School of Medicine, St. George's University, Grenada, West Indies; ^2^Department of Surgery, Saint Barnabas Medical Center, Livingston, NJ, USA; ^3^Department of Pathology, Saint Barnabas Medical Center, Livingston, NJ, USA; ^4^Department of Radiation Oncology, Saint Barnabas Medical Center, Livingston, NJ, USA; ^5^Department of Medical Oncology, Saint Barnabas Medical Center, Livingston, NJ, USA; ^6^Department of Surgery, University of Medicine and Dentistry of New Jersey (UMDNJ), Newark, NJ, USA

## Abstract

Myeloid Sarcoma (MS), a rare extra hematopoietic carcinoma composed of blast cells, is located primarily in extramedullary sites such as skin, soft tissue, lymph nodes, and bone. MS usually presents in the setting of coexisting acute myeloid leukemia (AML) and myeloproliferative disorders. Gastrointestinal involvement (GI) is extremely rare from nonspecific abdominal symptoms to obstruction. Eight cases of myeloid sarcoma involving the duodenum including the current case have been reported, overall mean age being 40 years (range 17–71) and M : F ratio 7 : 1. The prognosis of patients with *de novo* MS cases has been reported to be better than those who have a coexisting leukemia. MS is a rare extramedullary tumor, which should be considered in the differential diagnosis of a soft tissue mass involving the duodenum, especially if there is a coexisting hematological disorder. *De novo* cases often progress to AML, and current therapy involves Daunorubicin- and Cytarabine-based chemotherapy. The wide cytogenetic and molecular heterogeneity of MS implies a potential role for more targeted MS therapies, which may offer a curative strategy.

## 1. Introduction

Myeloid sarcomas (MS) are rare and potentially destructive extramedullary tumors consisting of immature myeloid cells that most often present in the skin, soft tissues, bone, and lymph nodes [1, 2]. Although MS was first described in 1911 by Burns, it has come to be referred to by many names [3]. The name “chloroma” was termed by King (1953), when he described multiple tumors with green color secondary to the presence of myeloperoxidase [4]. MS was coined “granulocytic sarcoma” by Rappaport, when he described tumors made up of granulocytes [5]. Today myeloid sarcoma is the preferred pathological term to describe tumors composed primarily of blast cells. These terms are also more reflective of the fact that many of the tumors are not green and have a white or pink color depending on their state of oxidation.

Many MS patients (if not most) have either a coexisting acute myeloid leukemia (AML), myeloproliferative, or myelodysplastic disorder at the time of diagnosis, or it appears at the first sign of relapse from one of these disorders. In rare cases, MS occurs *de novo* with no evidence of bone marrow involvement as seen in the current case. We report the case of a *de novo* MS presenting as a compressive mass involving the 3rd portion of the duodenum in a 48-year-old man who presented with nausea and vomiting. A comprehensive review of the literature with similar clinical presentation, diagnosis, management, and prognosis of patients with these rare GI *de novo* tumors are discussed.

## 2. Case Report

**Figure 1:**
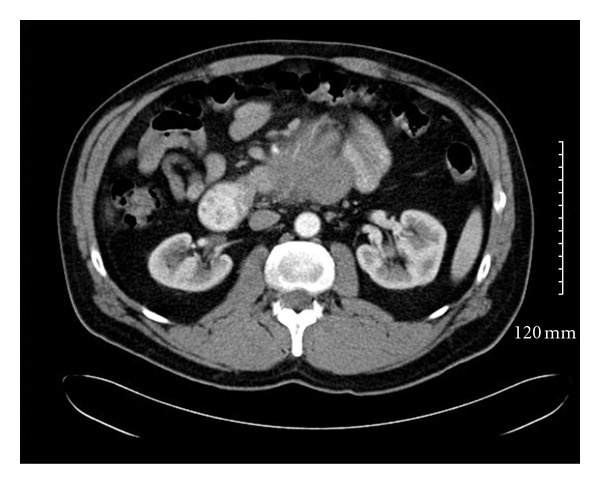
Initial CT abdomen with contrast showing a diffuse 6.1 cm × 5.9 cm × 8 cm thickening of the wall of duodenum with adjacent encasement of the superior mesenteric artery.

**Figure 2:**
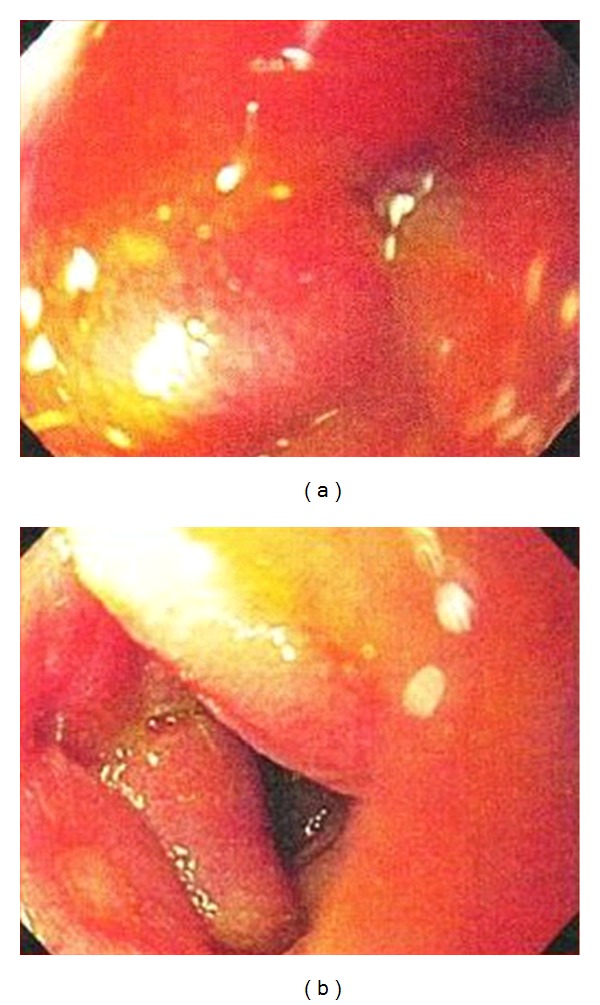
Esophagogastroduodenoscopy image on the left shows edematous and eythematous mucosa of the 3rd portion of the duodenum. Image on the right shows an area of 5 mm of ulceration in the 4th portion of the duodenum.

**Figure 3:**
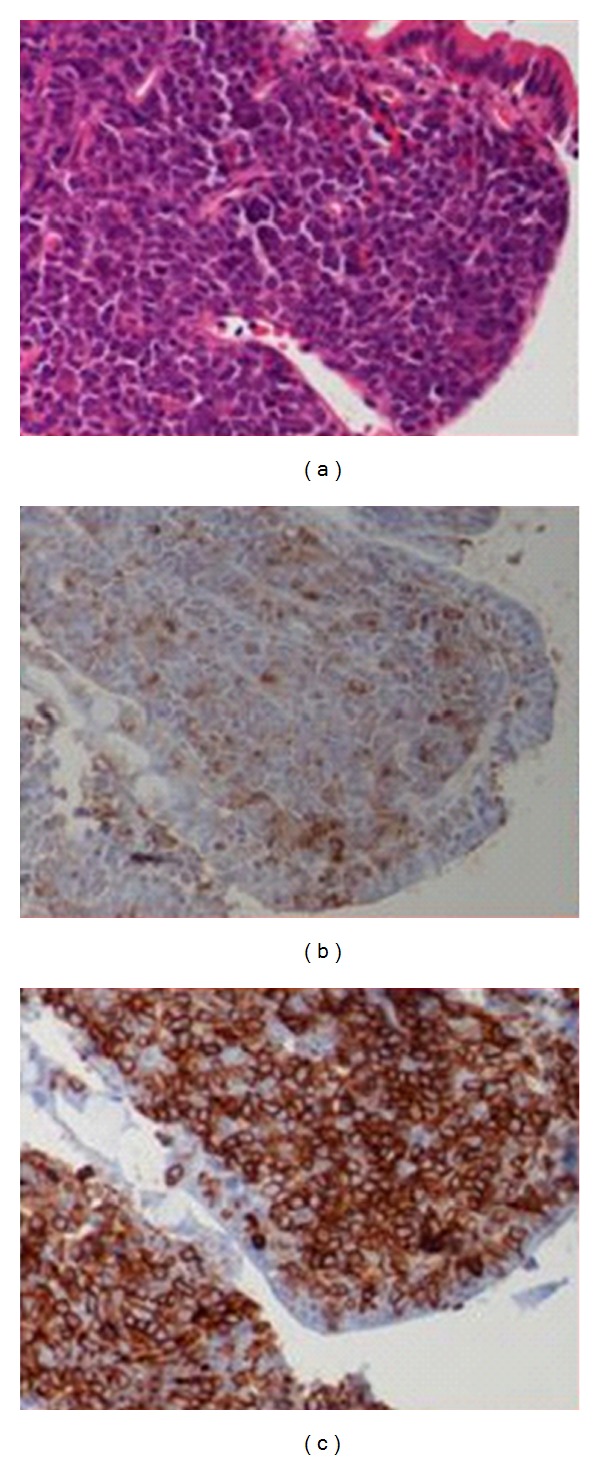
(a) Duodenal biopsy showing infiltration of blastoid cells into the mucosa. (H&E original magnification, ×400). (b) Myeloperoxidase (MPO) staining reveals same population of cells staining positive for MPO (original magnification, ×400). (c) Tumor cells staining bright yellow (positive) for CD34 (original magnification, ×400). (Image courtesy of Dr Mu Su).

 A 48-year-old man presented to the emergency room at Saint Barnabas Medical Center in Livingston, NJ, USA, complaining of nausea and colicky nonradiating epigastric pain of a 2-weeks duration that was not associated with food ingestion. He also reported intermittent constipation over the past two weeks. Past medical history was significant for bipolar disorder that was well controlled on Lithium. General physical examination revealed no abnormalities; the patient was anicteric and had no palpable lymphadenopathy. The abdominal examination revealed a mildly distended abdomen with moderate tenderness over the epigastrium. No rebound tenderness or guarding was present. Bowel sounds were normal and no organomegaly or masses were noted. Laboratory evaluation, including complete blood count, liver enzymes, renal function, and electrolytes, was all within normal limits. A computed tomography (CT) scan of the abdomen showed concentric wall thickening of the 3rd and 4th portions of the duodenum with adjacent soft-tissue encasement of the superior mesenteric artery and prominent mesenteric lymph nodes. The mass measured 6.1 cm × 5.9 cm × 8.0 cm (Figure 1). At that time, the differential diagnosis included lymphoma, carcinoma, or neuroendocrine tumor of the duodenum. An esophagogastroduodenoscopy (EGD) was performed, which revealed diffuse edematous and erythematous mucosa in the 3rd portion of the duodenum that extended to the 4th portion causing narrowing of the lumen. A small 5 mm area of ulceration in the 4th portion of the duodenum (Figure 2) was biopsied revealing diffuse infiltration of uniformly blastoid appearing cells completely occupying the mucosal space on hematoxylin and eosin (H&E) staining. Immunohistochemistry was positive for CD45 (weakly +), CD34 (+) (Figure 3), CD117 (+), CD33 (+), MPO (+), CD43 (bright +), and Bcl-2 (+). Ki-67 was highlighted in 70–80% of the cells. A peripheral blood smear revealed no circulating blasts, and the bone marrow aspirate showed no abnormal morphology in the cells present, and the percentages of blasts, promyelocytes, and granulocytes were within normal limits. Flow cytometry indicated no immunophenotypic evidence of hematolymphoid malignancy. Karyotype analysis of the bone marrow revealed a normal appearing 46, XY male complement. Although duodenal biopsy was consistent with myeloid sarcoma, additional tissue for cytogenetic studies was obtained via diagnostic laparoscopy. At biopsy, the tumor mass was found to encase the superior mesenteric artery and to be partially compressing both the 3rd and 4th portions of the duodenum. Chromosome testing from a laparoscopically obtained tissue from the base of the mesentery showed a chromosome complement with abnormal complex metaphase 47, XY male and a balanced translocation involving t(2,17) with breakpoints at 2q23 and 17q23. Numeric increases in chromosome 22 were also seen. A diagnosis of primary duodenal myeloid sarcoma without a coexisting hematologic disorder was confirmed, and treatment was initiated.

**Figure 4:**
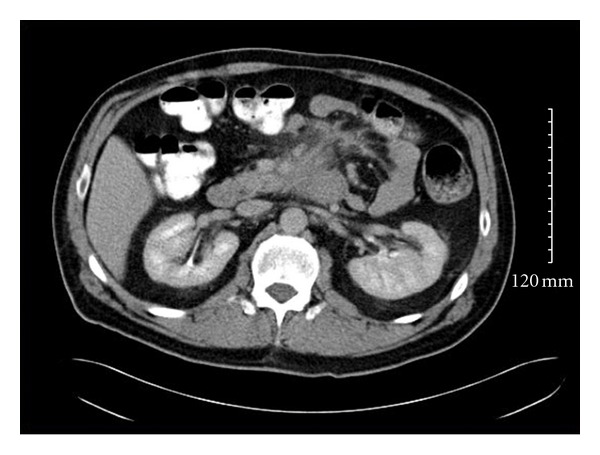
CT abdomen after 1 cycle of chemotherapy showing a decrease in size (2.9 cm × 3.5 cm × 4.4 cm tissue mass) in the distal duodenal mass.

The patient received 1 cycle of Cytarabine (1 g/m^2^) twice a day on days 1–3 and Idarubicin (12 mg/m^2^) on days 4, 5, and 6. Treatment was tolerated well, with rapid resolution of abdominal pain and obstructive symptoms. A follow up abdominal CT scan done two months later revealed a residual 2.9 cm × 3.5 cm × 4.4 cm tissue mass in the distal duodenum that was notably smaller than on original CT. There was no evidence of bowel obstruction (Figure 4). Following chemotherapy, patient underwent a second-look laparoscopy, which revealed a large area of treatment, related effect and multiple biopsies were obtained. Frozen-section analysis revealed no clear blast cells, and final histology demonstrated necrotic mesothelial lining fibroadipose tissue and single-fragment of fibrous tissue with chronic inflammation. There was no morphologic evidence of residual MS or definitive leukemia cells. Given the improvement seen, the patient was placed on high-dose Cytarabine (HiDAC) at 1.5 g/m^2^ as consolidation therapy. Patient also received tomotherapy 2400 cGy (12 fractions). At the time of report the patient remained symptom-free and has shown no evidence of additional hematological involvement after 18 months.

## 3. Materials and Methods

A comprehensive English search for all articles pertinent to myeloid sarcoma was conducted using PubMed, a search engine provided by the U.S. National Library of Medicine and the National Institutes of Health. Key words searched included myeloid sarcoma, duodenal myeloid sarcoma, gastrointestinal myeloid sarcoma, granulocytic sarcoma duodenum, and duodenal AML. Cases identified were analyzed according to age, gender, symptoms, site of involvement, associated hematologic malignancy, karyotype and cytogenetics, and treatment and patient prognosis.

## 4. Results

Eight cases of myeloid sarcoma with duodenal involvement including the current case have been reported since 1998. The clinical data, treatment, and patient prognosis in these cases are detailed in Table 1. In this group, 7 patients were male and 1 was female (M : F ratio 7 : 1). Overall mean age was 40 years (range 17–71). Three cases including the current one presented as *de novo* MS (37.5%); however, 1 patient in this group eventually developed AML at the time of report (12.5%). Six cases presented with MS were limited to the duodenum (75%), while two cases involved additional structures (25%). The prognosis of patients with *de novo* MS has been reported to be better than those who have a coexisting leukemia, which was also seen in the reported duodenal MS cases. Both patients with *de novo* MS who remained leukemia-free after treatment remained in remission, while 50% of those with leukemia eventually expired, 1 remained in remission (25%), and the outcome of one patient was not reported.

## 5. Discussion 

Myeloid sarcoma is a rare extramedullary malignant tumor composed of immature myeloid cells and can be identified by the type of blast cells that compose the mass (granulocytic, monocytic, and erythroid). The World Health Organization (WHO) has further classified granulocytic sarcomas into 3 main types based on the degree of maturation of the tumor: blastic (myeloblasts), immature (myeloblasts and promyelocytes), and differentiated (promyelocytes and more mature myeloid cells) [17]. These tumors are often associated with AML, CML, myeloproliferative, and myelodysplastic disorders either at the initial diagnosis, or at relapse of those diseases [18]. 

 MS has a predilection for males (2 : 1) but no apparent age preference as all age groups are represented in the literature [1]. MS has been reported in 2.5%−9.1% of cases of patients with AML, and the prevalence is estimated at 2/1,000,000 in adults and 0.7/1,000,000 in children, although these sarcomas are likely under diagnosed [12, 19]. 

 MS may occur in a variety of locations; however, the most common sites are skin (13%–22%), bone/spine (9%–25%), and lymph nodes (15%–25%), and less often the central nervous system and the orbits [12]. Gastrointestinal (GI) involvement has been infrequently reported. In a series of 62 MS cases, Neiman et al. found that overall GI involvement occurred only in 7% of the cases [19]. In a study of 72 MS GI cases, Yamauchi et al. found the prevalence of small bowel involvement to be 15% with no duodenal cases reported [20]. Other reported sites in the GI tract include the stomach, liver, pancreas, ileum, jejunum, appendix, and rectum. These patients presented with variable symptoms including abdominal pain, anorexia, obstruction, and in some cases bleeding due to perforation depending on the location of the tumor [13]. Duodenal MS can present with a wide spectrum of symptoms as well, including generalized abdominal pain, constipation, nausea, anorexia, jaundice, diarrhea, obstruction, or even perforation depending on the portion of duodenum involved and whether associated structures such as the bile duct has been compromised [11]. 

46%–75% of patients with isolated MS are initially diagnosed with other conditions such as non-Hodgkin's lymphoma [13, 21]. Pileri et al. noted that 10 of 25 cases of *de novo* MS were initially diagnosed as diffuse large B-cell lymphoma, small lymphocytic lymphoma, peripheral T-cell lymphoma, T-cell precursor lymphoma, and myeloid metaplasia when adequate immunohistochemistry studies were not performed [1].  Table 2 details the comparison between the clinical features seen in myeloid sarcoma, carcinoid tumors, lymphoma, and gastrointestinal stromal tumors (GIST). Radiologically, MS may mimic lymphomas due to the uniform contrast enhancement on CT; however, other diagnoses for a duodenal mass on CT also include neuroendocrine tumors and adenocarcinoma [6]. On histological examination, MS typically shows a diffuse and infiltrative population of myeloblasts and granulocytes on H&E staining. The neoplastic cells usually contain scant cytoplasm with large round-oval nuclei [22]. Immunohistochemistry offers one of the best methods in establishing the diagnosis of MS [18, 22]. Positive staining for markers MPO, CD34, CD117, and CD68 and lysozyme help identify the myeloid neoplastic cells [8, 22]. The chromosomal abnormalities that have been reported in conjunction with these extramedullary sarcomas include t(8,21), inv(16), t(9,11), 11q23, del(16q), and trisomy 8 [22, 23]. Interestingly, the patient presented in this paper did not have any of these listed abnormalities; however, he did have a balanced translocation involving chromosomes 2 and 17, which to our knowledge has not been reported in association with myeloid sarcomas. In a review of 20 GI MS cases, Zhang et al. noted that the inv(16) was often associated with intestinal cases [13]. Our patient did not have this abnormality. Standard investigation used in the workup of a duodenal mass was employed here, which included upper GI endoscopy, CT of the abdomen, and biopsy of the mass with H&E staining and immunohistochemistry.

 Although there is no clear consensus or guidelines on how to treat isolated MS, delays in treatment almost always progress to AML [21, 22]. The median time to the development of acute leukemia in the setting of isolated MS tumors has ranged from 5 to 12 months [13, 19]. A result therapy which should be instituted promptly and typically involves standard Daunorubicin- and Cytarabine-based chemotherapy for AML, which includes a 2-part-induction phase (to achieve complete remission) and a consolidation phase (to maintain complete remission) [9, 22]. Low-dose radiation therapy (24 Gy) has been suggested in cases that may require debulking due to compression of vital structures or in cases where intensive chemotherapy has failed [22]. Surgery is not the primary modality of treatment for MS but is indicated in cases of obstruction, perforation, or compression of vital structures [11]. The therapeutic response does not seem to be influenced by whether the tumor presents *de novo* or in conjunction with AML or other hematologic entity even when age, sex, and anatomical site are taken into consideration. Patients who undergo either allogenic or autologous bone marrow transplants seem to have a higher probability of survival and complete remission [1]. The median survival of MS patients without AML has been reported to be 36 months, while those progressing to AML have a poor prognosis with median survival between 6 and 14 months [20]. However given the rarity of MS, survival outcomes have been reported in only a handful of reports, and no control studies exist. The wide cytogenetic and molecular heterogeneity of the disease has prompted the use of targeted therapies (TK inhibitors, C-*KIT* inhibitors Imatinib and Dasatinib, and monoclonal antibodies) aimed at inhibiting the pivotal pathways of leukemogenesis and restoring normal hemopoiesis. In the future, a combination of different new drugs along with conventional chemotherapy may represent a more effective and potentially curative strategy [24]. 

## 6. Conclusion

Diagnosing duodenal MS can be a diagnostic challenge especially in patients with no previous leukemic disease. The symptoms related to the tumor mass are most often nonspecific, with abdominal pain sometimes being the only complaint, and as a result a wide variety of differential diagnoses are often entertained. In the evaluation of a solid GI mass, myeloid sarcoma should be considered in the differential diagnosis with confirmation by biopsy and immunohistochemical analysis of the tumor. Patients with MS should be thoroughly tested for the presence of concurrent leukemias or other myelodysplastic disorders. The rapid initial diagnosis of MS and immediate treatment with either chemotherapy, radiation, surgery, or a combination of any of these modalities are associated with a better prognosis, as misdiagnosis or treatment delays can result in disease progression to AML. Further studies linking cytogenic abnormalities with the location of the tumors may permit more targeted therapies.

## Figures and Tables

**Table 1:** Published reports of primary duodenal myeloid sarcomas (1998–2010).

Case report	Sex, age	Site	Associated malignancy	Karyotype/ cytogenetics	Treatment	Prognosis
Kim 1998 [6]	Male, 57	Duodenum	*De novo *	NR	Daunorubicin + Cytosine Arabinoside	Remission 7 months with no leukemia
Goor et al., 2003 [7]	Male, 27	Duodenum	NR, 30% blasts	Inv (16)	Induction (Cytarabine + Idarubicin), consolidation (high dose Cytarabine), allo BMT	Remission 2 years
Choi et al., 2007 [2]	Male, 71	Duodenum	CML	NR	NR	NR
Derenzini et al., 2008 [8]	Male, 40	Stomach (fundus, body), duodenum	*De novo*, AML 20 days later	45, XY	Induction (Ara-C, Etoposide, Idarubicin, 2nd induction (Ara-C, Idarubicin) after clinical relapse, allo BMT	Expired 50 days post BMT
Ghafoor et al., 2010 [9]	Male, 17	Duodenum	AML-M1	NR	2 courses induction (Ara-C, Daunorubicin, Etoposide), consolidation MACE (Amsacrine, Cytosine, Etoposide), + MIDAC (Mitoxantrone, high dose Cytarabine)	In remission 25 months post treatment
Jeong et al., 2010 [10]	Male, 35	Duodenum jejunum, left sternocleid-omastoid	AML	46, XY	Induction (Mitoxantrone, Etoposide, Cytarabine)	Expired due sepsis after chemotherapy
Antic et al., 2010 [11]	Female, 28	Duodenum	Primary, AML 2 months later	46, XX	Patient refused treatment	NR

NR: not reported; BMT: bone marrow transplant.

**Table 2:** A comparison between common clinical presentations of myeloid sarcomas, carcinoid, lymphoma, and gastrointestinal stromal tumors (GIST).

	Myeloid sarcoma [1, 11–13]	Carcinoid [14]	Lymphoma [15]	GIST [16]
General characteristics	Extramedullary involvement	Indolent tumor that originate in cells of the neuroendocrine system that may produce hormones	Hodgkin's and Non-Hodgkin varieties involving lymphocytes of B, T, or NK cell lineage	Submucosal mesenchymal neoplasms of the GIT
Incidence (cases/million persons/year)	2 (adults) 0.7 (children)	20	Hodgkin 12 (<20 yrs) NHL 19 (female 20–24) 29 (male 20–24) 390 (female 60–64) 547 (male 60–64)	10–20
Male : female ratio	2 : 1	No preference	NHL−1.4 : 1, ratio varies with subtype	No clear preference although some studies indicate higher male incidence
Anatomic location	Skin, soft tissues, bone, lymph nodes, orbits, and CNS.	Multiple locations. GI carcinoids found in appendix, small intestine, rectum, colon, gallbladder, and kidney	Lymph nodes. Extranodal sites: skin, brain, bowel, bone, and thymus	50%–70% stomach, 20%–30% small intestine, 5%–15% colon/rectum, esophagus (<5%), rare in omentum and mesentery
Symptoms at presentation	Dependent on location of tumor. GI symptoms may range from nonspecific to jaundice or obstruction	Duodenal carcinoids may present with nausea, vomiting, abdominal pain, and hemorrhage due to excess gastrin production	Palpable painless lymph nodes, chest pain, constitutional (B) symptoms, and fatigue	Asymptomatic or nonspecific abdominal symptoms such as obstruction, appendicitis-like pain, and acute abdomen due to tumor rupture.
Pathology	Diffuse and infiltrative population of myeloblasts and granulocytes. The neoplastic cells usually contain scant cytoplasm with large round-oval nuclei	Firm white, yellow, or gray nodules. Neuroendocrine cells have uniform nuclei and abundant granular or faintly staining (clear) cytoplasm	Hodgkin: Reed-Sternberg cells NHL: varies depending on type	Range from slow growing, indolent to aggressive malignant cancers
Immunohistochemistry	MPO, CD34, CD117, CD68, and lysozyme	No specific IHC. May test for levels of 5-HIAA, CgA	Varies depending on type: CD30, CD15, CD5, CD10, and TdT	CD117, CD34
Prognosis	The median survival of MS patients without AML has been reported to be 36 months, while those progressed to AML have a poor prognosis with median survival between 6 and 14 months	Dependent on site, size, and anatomical extent of disease. Expression of Ki-67 and p53 may be associated with poor prognosis	5-year survival ranges from 60%–82% depending on stage and type	Important factors are size of tumor and mitotic rate, average 5 yr survival 30%–60%. Duodenal GIST, 2 cm low risk >10 cm high risk. Mitotic risk >5 per 50 hpf

Abbreviations: GIST: gastrointestinal stromal tumors; CD: cluster of differentiation; CgA: chromogranin A; TdT: terminal deoxynucleotidyl transferase; NHL: non-Hodgkin lymphoma; hpf: high power field; CNS: central nervous system; GI: gastrointestinal; MS: myeloid Sarcoma; MPO: myeloperoxidase; HIAA: Hydroxy Indole Acetic Acid; NK: natural killer; IHC: immunohistochemistry.

## References

[1] Pileri SA, Ascani S, Cox MC (2007). Myeloid sarcoma: clinico-pathologic, phenotypic and cytogenetic analysis of 92 adult patients. *Leukemia*.

[2] Choi EK, Ha HK, Park SH (2007). Granulocytic sarcoma of bowel: CT findings. *Radiology*.

[3] Burns A (1811). *Observation of Surgical Anatomy, Head and Neck*.

[4] King A (1853). A case of chloroma. *Monthly Journal of the Medical Society*.

[5] Rappaport H (1966). Tumours of the hematopoietic system. *Atlas of Tumor Pathology, Section III, Fascicle 8. Armed Forces Institute of Pathology*.

[6] Kim H (1998). Primary granulocytic sarcoma of the duodenum: radiologic and endoscopic findings. *American Journal of Roentgenology*.

[7] Goor O, Goor Y, Konikoff F (2003). Epigastric distress caused by a duodenal polyp: a rare presentation of acute leukemia. *Journal of Clinical Oncology*.

[8] Derenzini E, Paolini S, Martinelli G (2008). Extramedullary myeloid tumour of the stomach and duodenum presenting without acute myeloblastic leukemia: a diagnostic and therapeutic challenge. *Leukemia and Lymphoma*.

[9] Ghafoor T, Zaidi A, Al Nassir I (2010). Granulocytic sarcoma of the small intestine: an unusual presentation of acute myelogenous leukaemia. *Journal of the Pakistan Medical Association*.

[10] Jeong SH, Han JH, Jeong SY (2010). A case of donor-derived granulocytic sarcoma after allogeneic hematopoietic stem cell transplantation. *The Korean Journal of Hematology*.

[11] Antic D, Elezovic I, Bogdanovic A (2010). Isolated myeloid sarcoma of the gastrointestinal tract. *Internal Medicine*.

[12] Breccia M, Mandelli F, Petti MC (2004). Clinico-pathological characteristics of myeloid sarcoma at diagnosis and during follow-up: report of 12 cases from a single institution. *Leukemia Research*.

[13] Zhang XH, Zhang R, Li Y (2010). Granulocytic sarcoma of abdomen in acute myeloid leukemia patient with inv(16) and t(6;17) abnormal chromosome: case report and review of literature. *Leukemia Research*.

[14] http://www.cancer.gov/cancertopics/pdq/treatment/gastrointestinalcarcinoid/HealthProfessional.

[15] http://www.lls.org/#/diseaseinformation/getinformationsupport/factsstatistics/hodgkinlymphoma/.

[16] http://www.cancer.gov/cancertopics/pdq/treatment/gist/HealthProfessional.

[17] Jaffe ES, Harris NL, Stein H (2001). *World Health Organization Classification of Tumours—Tumours of Haematopoietic and Lymphoid Tissues*.

[18] Sivan-Hoffmann R, Waksman I, Cohen HI (2011). Small bowel obstruction as a presenting sign of granulocytic sarcoma. *The Israel Medical Association Journal*.

[19] Neiman RS, Barcos M, Berard C (1981). Granulocytic sarcoma: a clinicopathologic study of 61 biopsied cases. *Cancer*.

[20] Yamauchi K, Yasuda M (2002). Comparison in treatments of nonleukemic granulocytic sarcoma: report of two cases and a review of 72 cases in the literature. *Cancer*.

[21] Meis JM, Butler JJ, Osborne BM, Manning JT (1986). Granulocytic sarcoma in nonleukemic patients. *Cancer*.

[22] Bakst RL, Tallman MS, Douer D (2011). How I treat extramedullary acute myeloid leukemia. *Blood*.

[23] Jenkins CI, Sorour Y (2005). Case report: a large extramedullary granulocytic sarcoma as the initial presenting feature of chronic myeloid leukemia. *MedGenMed*.

[24] Ferrara F (2012). New agents for acute myeloid leukemia: is it time for targeted therapies?. *Expert Opinion on Investigational Drugs*.

